# Supplementary material for: Comparative multiomics analysis of cell physiological state after culture in a basket bioreactor
Source: Sci Rep. 2022 Nov 23;12:20161. doi: 10.1038/s41598-022-24687-4 (PMC9686226; doi:10.1038/s41598-022-24687-4)
Supplement: Supplementary file 1 — Supplementary Information 1. [file 41598_2022_24687_MOESM1_ESM.zip › raw data/Transcriptomic raw data/7.AS/2.dasplot/TE1vsTC1/RI/19_EXOC3L1_NW_023666047.1_23024150_23024274_+@NW_023666047.1_23024150_23024581_+@NW_023666047.1_23024356_23024581_+.pdf]

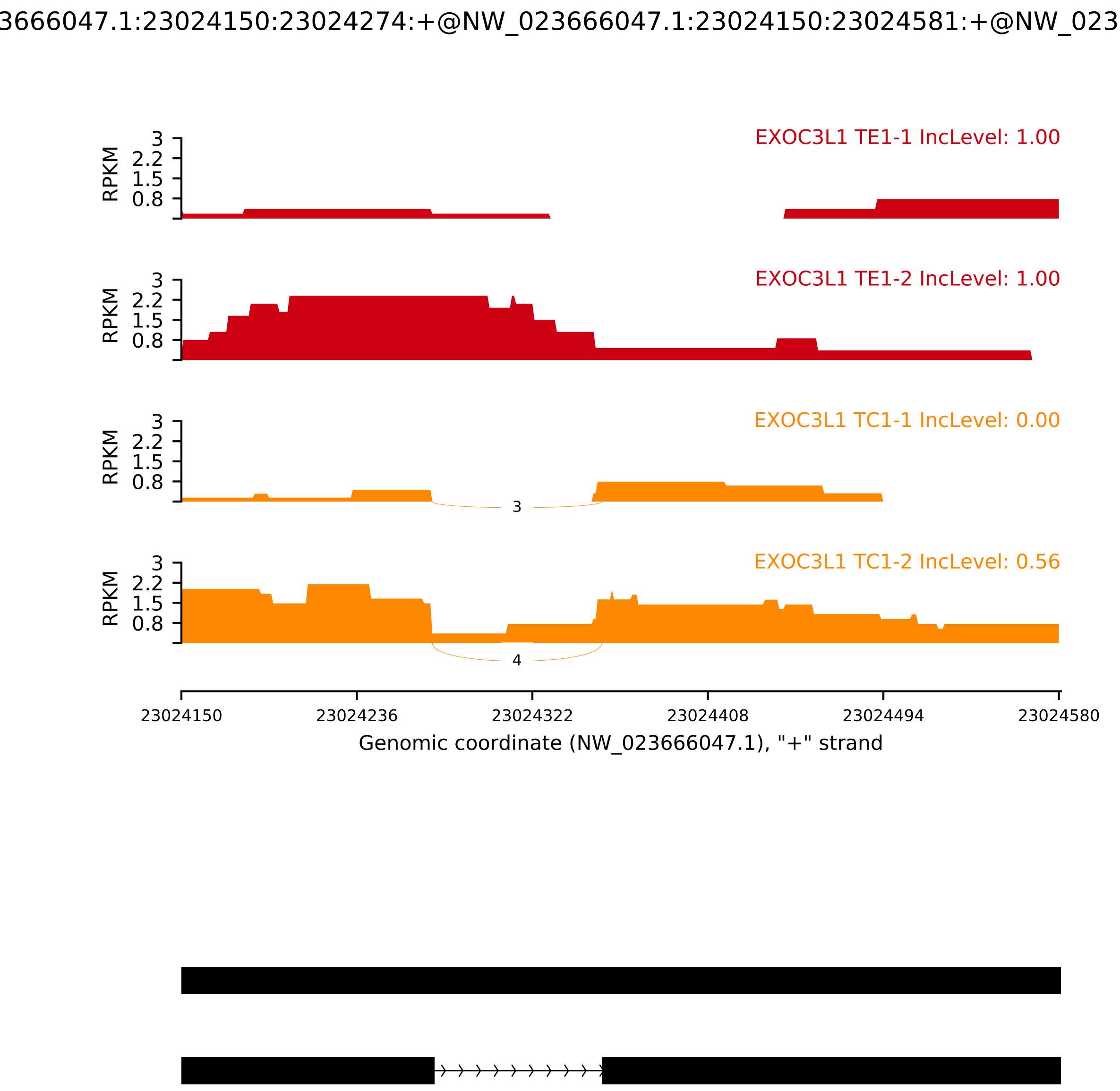

3666047.1:23024150:23024274:+@NW_023666047.1:23024150:23024581:+@NW_023666047.1:23024356:2302
EXOC3L1 TE1-1 IncLevel: 1.00
EXOC3L1 TE1-2 IncLevel: 1.00
EXOC3L1 TC1-1 IncLevel: 0.00
EXOC3L1 TC1-2 IncLevel: 0.56
RPKM
3
2.2
1.5
0.8
3
4
23024150
23024236
23024322
23024408
23024494
23024580
Genomic coordinate (NW_023666047.1), "+" strand
